# Fractal Analysis of Human Gait Variability via Stride Interval Time Series

**DOI:** 10.3389/fphys.2020.00333

**Published:** 2020-04-15

**Authors:** Angkoon Phinyomark, Robyn Larracy, Erik Scheme

**Affiliations:** ^1^Institute of Biomedical Engineering, University of New Brunswick, Fredericton, NB, Canada; ^2^Department of Electrical and Computer Engineering, Faculty of Engineering, University of New Brunswick, Fredericton, NB, Canada

**Keywords:** box-counting dimension, detrended fluctuation analysis, gait analysis, gait variability, Higuchi's fractal dimension, hurst exponent, nonlinear dynamics, stride interval time series

## Abstract

Fractal analysis of stride interval time series is a useful tool in human gait research which could be used as a marker for gait adaptability, gait disorder, and fall risk among patients with movement disorders. This study is designed to systematically and comprehensively investigate two practical aspects of fractal analysis which significantly affect the outcome: the series length and the parameters used in the algorithm. The Hurst exponent, scaling exponent, and/or fractal dimension are computed from both simulated and experimental data using three fractal methods, namely detrended fluctuation analysis, box-counting dimension, and Higuchi's fractal dimension. The advantages and drawbacks of each method are discussed, in terms of biases and variability. The results demonstrate that a careful selection of fractal analysis methods and their parameters is required, which is dependent on the aim of study (either analyzing differences between experimental groups or estimating an accurate determination of fractal features). A set of guidelines for the selection of the fractal methods and the length of stride interval time series is provided, along with the optimal parameters for a robust implementation for each method.

## 1. Introduction

Gait analysis has been generally studied using traditional linear analysis methods, adopting biomechanical models in which variability was not of interest (e.g., comparing the means of spatio-temporal gait parameters between groups). However, steady-state walking could be characterized by the presence of subtle variations observed in stride intervals (the time period between consecutive initial contacts of the same foot). In other words, the variability of stride intervals (or gait variability) could be treated as a meaningful and interpretable metric of gait. More advanced, nonlinear analysis methods, derived from chaos theory, have therefore been proposed to analyze the temporal organization of gait variability (Chau, 2001).

While many methods of fractal analysis exist to explore long-range autocorrelations in stride interval time series, detrended fluctuation analysis (DFA) has been the most commonly used. This may be because DFA was used by the pioneering works of Hausdorff et al. (1996) and provides reasonably accurate results for short time series as compared to other classical methods for estimating the Hurst exponent (Delignieres et al., 2006). Specifically, scaling exponent-like quantities, including the DFA's scaling exponent and the Hurst exponent, show normal human walking produces persistent stride time fluctuations and a drift toward randomness is observed with aging and neurological disorders (Hausdorff et al., 1997, 2000). As a result, these measures could be used as a marker for gait adaptability, gait disorder, and fall risk among patients with movement disorders, such as Parkinson's disease (PD), amyotrophic lateral sclerosis (ALS), and Huntington's disease (HD).

Although DFA has become the de facto standard for analyzing statistical persistence in gait data, it too is sensitive to the length of the time series, which remains a significant limitation. Previous studies using simulated and/or experimental signals have shown that estimation accuracy is directly related to the series length, and lengths of around 500–600 data points or less, corresponding to the number of stride intervals from approximately 10–12 min of self-paced walking, yield questionable results due to the high bias and variance in the estimation of the scaling exponent (Damouras et al., 2010; Marmelat and Meidinger, 2019). Some studies have even suggested that DFA cannot give reliable results with time series shorter than 4,096 data points (Eke et al., 2002). Such relatively long recordings, however, are difficult to collect from older adults or clinical populations, and thus the vast majority of previous studies have collected stride interval time series in these populations for only 2–5 min (i.e., between 100 and 250 strides) (Moon et al., 2016). The results from such studies should therefore be interpreted with caution.

Besides the length of time series under consideration, another practical aspect of DFA that significantly affects the analysis outcome is the set of parameters used in the algorithm: the box size range, the box size increment, and the order of polynomial fits for a detrending operation. Unfortunately, the implementation of DFA to stride interval time series is not yet standardized and varies considerably between studies, in which decisions are usually made on an *ad hoc* or subjective basis. Some previous studies have investigated these practical aspects, but have typically studied them in isolation (e.g., only impact of series length, or only one of the DFA parameters such as box size range). In addition, Warlop et al. (2017) found that using more robust methods (such as evenly-spaced box size increments) could provide reliable results for shorter time series (256 instead of 512 data points). To the best of our knowledge, however, a systematic and comprehensive investigation of the length of stride interval time series and all the DFA parameters has never been completed. Thus, a better understanding of interactions between the two practical aspects of DFA that affect the analysis outcome, is warranted.

As the DFA's scaling exponent and the Hurst exponent have been found to be unreliable for very short time series (<250 strides), another approach to explore fractal dynamics of gait has recently been proposed. Dierick et al. (2017) deployed a box-counting dimension (BC) algorithm to compute fractal dimension and used it as an indicator of complexity during walking. The results showed that walking forward exhibited maximal complexity whereas non-standard, but not pathological, walking (such as walking backward or individuals with galvanic vestibular stimulation) had lower complexities. Interestingly, no linear relationship between fractal dimension and scaling exponent was found, which is in contrast with previous studies which have implicitly considered the fractal dimension and the scaling exponent to be directly related. This may be due to the limitations of the methods or the parameters used (e.g., an under/overestimation bias). Chakraborty et al. (2015) used a Higuchi's fractal dimension (HG) algorithm to explore the difference between normal walking and dual tasking (performing a secondary task while walking) for healthy controls and PD subjects. Unfortunately, an appropriate value of the interval time parameter was not determined, and thus no hypothesized differences were found. Similar to classical Hurst exponent methods, including DFA, the choice of parameters used has a significant impact on the value of fractal dimension, and the subsequent conclusions. Therefore, it is necessary to identify an appropriate value of these parameters. Unlike Hurst exponent methods, fractal dimension methods can be calculated for a wide range of motions, not only fractional Brownian motions, and their accuracy is less influenced by the length of the time series (Sánchez-Granero et al., 2012).

Therefore, the first purpose of this study was to identify a robust implementation of these fractal methods for short time series. A systematic and comprehensive investigation of all the parameters was performed for DFA, BC, and HG using simulated exact fractal series. Based on these robust implementations, the second purpose of this study was to compare the estimation accuracies of the three fractal methods in terms of biases and variability as well as to provide guidelines for the selection of the length of stride interval time series. The third purpose of this study was to provide guidelines for the selection of the fractal methods to obtain an accurate determination of the exponent that characterizes the system under study and to analyze differences between experimental groups. It was hypothesized that (1) the optimal implementation of the fractal methods could reduce bias and variance in the estimation of Hurst exponent and fractal dimension, and could thus provide acceptable results for shorter time series; (2) fractal dimension methods (i.e., BC and HG) could provide more accurate results than the Hurst exponent method (i.e., DFA), especially for short time series, and thus could be used as a more efficient solution to study human gait alterations with aging and disease; and (3) normal human walking produces persistent stride time fluctuations and a drift toward randomness is observed with aging and neurological disorders. We also speculate that both indexes (DFA's scaling exponent/Hurst exponent and fractal dimension) are related under the robust implementations.

## 2. Methods

### 2.1. Simulated and Experimental Data

To investigate the effect of series length (*N*) on the Hurst exponent (*H*), 100 simulated fractional Brownian motion (fBm) time series were generated for each of seven values of *N* (*N* being a power of 2; 2^*p*^) ranging from 16 (*p* = 4) to 1,024 (*p* = 10) as well as each of nine values of *H* ranging from 0.1 to 0.9 by steps of 0.1. Two fBm generators were used following the previously validated algorithms proposed by Abry and Sellan (1996) and Kroese and Botev (2015) to ensure the results were not dependent on the fBm algorithm. It is important to note that the use of 100 simulated series per condition was sufficient to show contrast between means (biases) and standard deviation (variability) (Hausdorff et al., 1997; Delignieres et al., 2006), and the purpose of this study was not to accurately determine the mathematical relationships between true *H* and estimated Ĥ.

For the experimental data, two databases, contributed by Hausdorff et al. (2000), were investigated: (1) gait in neurodegenerative disease database; and (2) gait in aging and disease database. The first dataset consists of stride interval time series from 15 patients with PD, 13 ALS, 20 HD, and 16 healthy control subjects. The second dataset includes stride interval time series from 5 healthy young adults, aged between 23 and 29 years old, 5 healthy older adults, aged between 71 and 77 years old, and 5 older adults, aged between 60 and 77 years old, with PD. The protocols for both datasets are similar. Briefly, subjects were asked to walk continuously at their normal pace on level ground either along a long hallway for 5–6 min for the patients and healthy controls or in a roughly circular path for 15 min for healthy subjects. The raw force signals were measured using force-sensitive insoles with a sampling rate of 300 Hz, and subsequently used to compute the stride interval time series (Hausdorff et al., 1995). A median filter was applied to remove outliers (during the walking turns) which were considered to be three standard deviations from the median value.

### 2.2. Detrended Fluctuation Analysis (DFA)

The Hurst exponent *H* is calculated from the rescaled range (R/S) analysis, using range *R* of the integrated fluctuations and rescaling by the standard deviation *S*, as the effect of window size is inspected. In a similar way, DFA inspects the root mean square (RMS) of the detrended and integrated fluctuations as a function of window size, where a detrending operation is performed to enable correct *H* estimation in the presence of extrinsic non-stationaries (Peng et al., 1995). The DFA algorithm consists of a pipeline of six stages:

1. A time series *x*(*t*) of total length *N* is first integrated. This integration process converts the time series into a random walk. The integrated series *y*(*j*), also called a cumulative sum or profile, is defined by:
  (1) $$\begin{array}{l} y\left(j\right)=\overset{j}{\underset{t=1}{\sum }}\left[x\left(t\right)-\bar{x}\right],\;j=1,\ldots ,N, \end{array}$$
  where $\bar{x}$ represents the mean value of the time series.
2. The integrated time series *y*(*j*) is divided into *L* non-overlapping time windows or boxes of length *n*.
3. Within each box, a least-squares fit (or a polynomial fit of order *k*) is applied to the series *y*(*j*). The coefficient of *y* coordinate is denoted by *y*_*n*_(*j*). Each least-squares line presents the semi-local trend in that box.
4. The RMS fluctuation of the detrended and integrated time series is calculated by:
  (2) $$\begin{array}{l} F\left(n\right)=\sqrt{\frac{1}{N}\overset{N}{\underset{j=1}{\sum }}{\left[y\left(j\right)-y_{n}\left(j\right)\right]}^{2}}. \end{array}$$
5. This computation (stages 2–4) is repeated over a range of different box sizes *n*.
6. The DFA's scaling exponent α is then computed as the slope of a straight line fit to the log-log graph of *F*(*n*) and *n*, and is simply an estimate of the Hurst exponent *H*. It should be noted that while α for fractional Gaussian noise (fGn) is equal to *H*, for fBm α is equal to *H* + 1.

The scaling exponent α can explain the behavior of the time series as follows:

- 0 < α <0.5 indicates anti-persistent long-term correlations, meaning that a large value is more likely to be followed by a small value and vice versa.
- α = 0.5 indicates the absence of long-term correlations (white noise).
- 0.5 < α <1.0 indicates persistent long-term correlations, meaning that a large value (compared to the mean) is more likely to be followed by a large value and vice versa.
- α ≃ 1.0 indicates 1/*f* noise (pink noise).
- α ≃ 1.5 indicates Brownian noise.

### 2.3. Box-Counting Dimension (BC)

While the Hurst exponent *H* is used as a measure of long-term autocorrelation of time series, the fractal dimension *D*—another index for characterizing fractal patterns—is used as a measure of complexity of the time series. One of the most popular fractal dimension methods is the BC algorithm, and consists of a pipeline of five stages:

1. A time series *x*(*t*) of total length *N* is divided into *L* non-overlapping time windows, or intervals, of length *n*.
2. Within each time window, the number of square boxes of size *n* required to cover the points is computed by:
  (3) $$b_{n}(j)=\left\lceil \frac{h}{n}\right\rceil =\left\lceil \frac{\max(x_{n})-\min(x_{n})}{n}\right\rceil ,$$
  where *h* represents the signal amplitude change (or the height) and *x*_*n*_ represents the time series within each time window *j*.
3. The total number of boxes required to cover the total curve at the resolution *n* is calculated by:
  (4) $$\begin{array}{l} B\left(n\right)=\overset{L}{\underset{j=1}{\sum }}b_{n}\left(j\right). \end{array}$$
4. These computations (stage 1–3) are then repeated over a range of different box sizes *n*.
5. The BC dimension *D*_*BC*_ is computed as the slope of a straight line fit to the log-log graph of *B*(*n*) and 1/*n*. It should be noted that for fBm, *D* is directly related to *H* where *D* = 2 − *H*. For time series, *D* typically lies between 1 (a differentiable curve) and 2 (a surface with a differentiable boundary).

### 2.4. Higuchi's Fractal Dimension (HG)

In addition to standard algorithms such as the BC, more effective methods have been developed, such as the methods by Higuchi (1988), Katz (1988), Petrosian (1995). The principle of the HG algorithm is to sum the change in amplitude *h* normalized to the time interval *n*, and is comparable to the BC algorithm, except with “boxes” of “non-integer” height. The scheme of the HG algorithm consists of a pipeline of four stages:

1. A time series *x*(*t*) of total length *N* is first used to construct a new time series $X_{n}^{m}$, which is defined by:
  (5) $$\begin{array}{l} X_{n}^{m}=\{x(m),x(m+n),x(m+2n),\ldots ,x\\ \;\left(m+\left\lfloor \frac{N-m}{n}\right\rfloor n\right)\},\;m=1,\ldots ,n, \end{array}$$
  where *m* represents the initial time and *n* indicates the time interval.
2. The length of the curve $X_{n}^{m}$ is calculated by:
  (6) $$\begin{array}{l} L_{m}(n)=\frac{1}{n}\{\left(\sum_{j=1}^{\left\lfloor \frac{N-m}{n}\right\rfloor }|X(m+jn)-X(m+(j-1)n)|\right)\\ \;\frac{N-1}{\left\lfloor \frac{N-m}{n}\right\rfloor n}\}. \end{array}$$
3. These computations (stage 1–2) are repeated over a range of different interval lengths *n*.
4. The fractal dimension *D*_*HG*_ is then computed as the slope of a straight line fit to the log-log graph of *L*_*m*_(*n*) and *n*.

The fractal dimension *D* measures the degree to which the curve fills out the plane, where a simple curve has dimension equal 1 and a plane has dimension equal 2. In other words, the fractal dimension indicates the amount of information required to describe the time series. The fractal dimension *D* also measures the complexity of the signal. The term “complexity” is associated with the temporal structure of the signal, which usually lies in an intermediate state between two non-physiological situations: absence of variability (e.g., pink noise, *D* ≃ 1.8; Brown noise, *D* ≃ 1.5) and unstructured randomness (e.g., white noise, *D* ≃ 2.0) (Gomolka et al., 2018).

### 2.5. Parameter Optimization

For DFA, five parameters were evaluated: (1) the minimum box size, *n*_*min*_, (2) the maximum box size, *n*_*max*_, (3) the increment method, (4) the increment factor, *d*, and (5) the polynomial order, *k*. The literature suggests several values for the box size range. For example, *n*_*min*_ can be 3, 4, or 10, and the *n*_*max*_ should be less than one-tenth (*N*/10), a quarter (*N*/4), or a half (*N*/2) of the series length (Hu et al., 2001; Ma et al., 2005; Abásolo et al., 2008; Phinyomark et al., 2011; Wallot et al., 2013). There are two different methods of incrementing between box sizes: (1) an arithmetic progression (AP), i.e., the increment of the box size is fixed to be equal to the increment factor (*d*) throughout the total length (*n*_*i*_ = *n*_*min*_ + (*i* − 1)*d*; *i* = 1, …, *L*); and (2) a geometric progression (GP), i.e., the box size is increased based on a power of two ($n_{i}=n_{min}\times 2^{\left(i-1\right)};i=1,\ldots ,L$). In addition to typical linear detrending (*k* = 1), second and third order polynomial fitting were also used to remove trends of higher order. Like DFA, the minimum and maximum box sizes and the method of incrementing between box sizes must be determined for the BC algorithm. For HG, only the maximum interval length *n*_*max*_ are optimized in this study, where *n*_*min*_ = 1 and the AP method of incrementing between interval lengths was used with *d* = 1. To summarize, all parameter options that were examined in this study are shown in Table 1. It should be noted that parameter names and their abbreviations were chosen to be as concise as possible while still translating across the fractal methods.

**Table 1:** A summary of parameter options studied for the three fractal methods: DFA, BC, and HG.

**Method**	**Parameter**	**Option**
DFA	Minimum box size (*n*_*min*_)	2, 4, 6, 8, 10
	Maximum box size (*n*_*max*_)	*N*/10, *N*/9, *N*/5, *N*/4, *N*/2
	Increment method	AP, GP
	Increment factor (*d*)	2, 4, 6
	Polynomial order (*k*)	1, 2, 3
BC	Minimum box size (*n*_*min*_)	2, 4, 6, 8, 10
	Maximum box size (*n*_*max*_)	*N*/10, *N*/9, *N*/5, *N*/4, *N*/2
	Increment method	AP, GP
	Increment factor (*d*)	2, 4, 6
HG	Maximum interval length (*n*_*max*_)	2, 4, 8, 16, 32, 64

### 2.6. Statistical Analysis

Two main characteristics: (1) bias and (2) variability were examined using simulated fBm time series with a range of identical true *H* exponents. To assess bias (the deviation of the estimated Ĥ from the theoretical *H*), several indicators were employed including the plot of mean Ĥ vs. *H*, the mean error (Ĥ − *H*), and the mean absolute error (|Ĥ − *H*|). To assess variability of estimations obtained from series of exact theoretical *H*, standard deviation of Ĥ was computed.

A box plot of experimental data was used to show the summary of scaling exponents and fractal dimensions including the minimum, first quartile, median, third quartile, and maximum. The mean and standard deviation were also reported. For statistical differences between multiple groups, the Kruskal–Wallis test—a nonparametric version of classical one-way analysis of variance (ANOVA)—was used. The Wilcoxon rank sum test—a non-parametric test for two independent populations—was used to compare between two groups at a time (*post-hoc* comparisons). Group differences were considered statistically different if *p* ≤ 0.05. Further, associations between fractal variables were evaluated by using Spearman's correlation coefficient ρ.

## 3. Results

**Figure 1:**
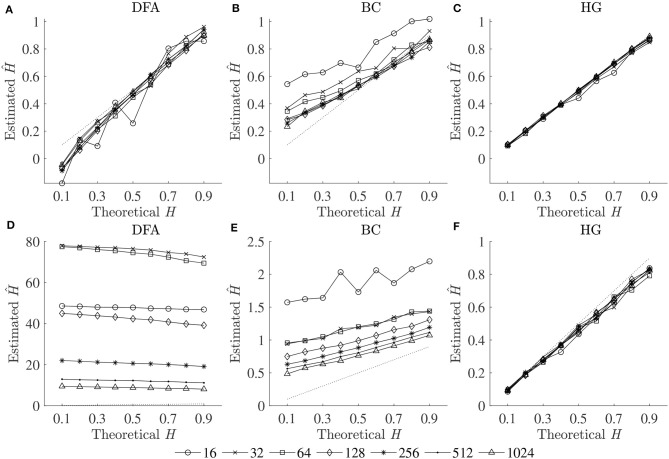
Plots of theoretical *H* and mean estimated Ĥ using the optimal parameters (upper panels) and the worst-case parameters (lower panels) for three fractal methods: **(A,D)** DFA, **(B,E)** BC, and **(C,F)** HG.

The optimal values of all fractal parameters for each series length were determined, in which the minimum estimation errors were found, and are shown in Table 2. It is important to note that the results for both fBm generators were the same. The optimization of parameters is important as illustrated in Figure 1 by comparing mean errors in the estimation of *H* using the optimal parameters (Table 2) and the worst-case parameters (out of all the options in Table 1; see Table S1).

**Table 2:** A summary of optimal values of all the parameters for three fractal methods: DFA, BC, and HG for each length of the time series.

**Method**	**Parameter**	**Option for each series length (**N**)**						
		**16**	**32**	**64**	**128**	**256**	**512**	**1024**
DFA	Minimum box size (*n*_*min*_)	4	4	4	4	4	4	4
	Maximum box size (*n*_*max*_)	*N*/2	*N*/2	*N*/2	*N*/2	*N*/5	*N*/9	*N*/10
	Increment method	AP	AP	GP	GP	AP	AP	AP
	Increment factor (*d*)	2	4	n/a	n/a	6	6	4
	Polynomial order (*k*)	1	2	2	2	2	2	1
BC	Minimum box size (*n*_*min*_)	4	6	4	8	4	4	4
	Maximum box size (*n*_*max*_)	*N*/2	*N*/2	*N*/2	*N*/2	*N*/2	*N*/2	*N*/4
	Increment method	AP	AP	AP	AP	GP	GP	AP
	Increment factor (*d*)	2	2	6	6	n/a	n/a	2
HG	Maximum interval length (*n*_*max*_)	4	4	4	8	4	4	4

**Figure 2:**
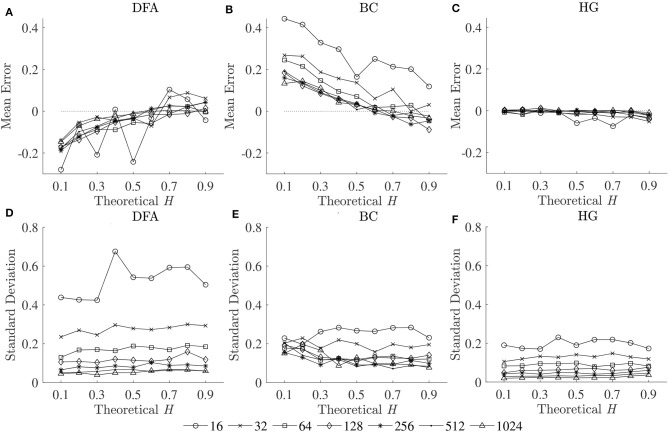
Mean errors (upper panels) and standard deviations (lower panels) of estimated Ĥ for three fractal methods: **(A,D)** DFA, **(B,E)** BC, and **(C,F)** HG.

**Figure 3:**
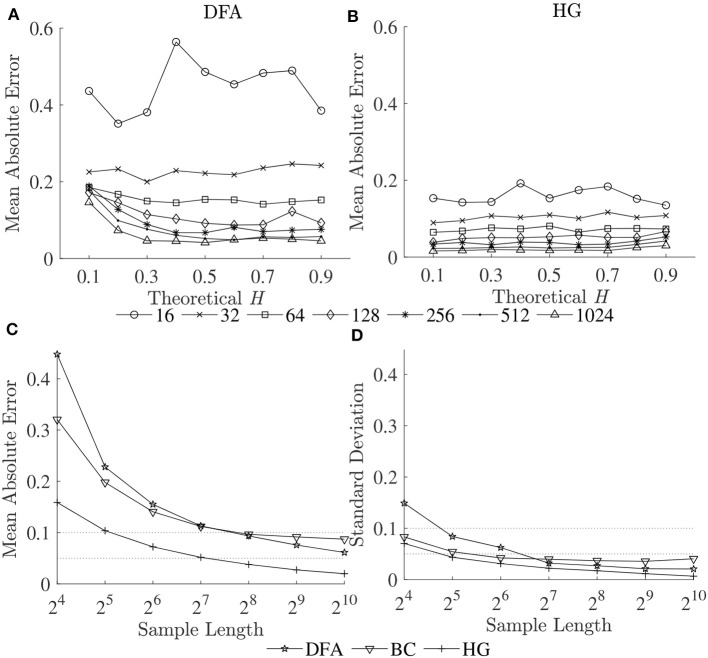
Mean absolute errors of estimated Ĥ vs. theoretical *H* for **(A)** DFA and **(B)** HG; and **(C)** mean absolute errors and **(D)** standard deviations of estimated Ĥ over a possible range (0.1–0.9) vs. sample length (16–1,024 data points).

To compare the estimation accuracies of the three fractal methods, the mean errors, and standard deviations of the estimated Ĥ were computed based on the robust implementation determined, and are shown in Figure 2. Since both HG and BC algorithms extracted fractal dimension, and HG could provide more accurate estimation than BC, *D*_*HG*_ was thus used to compare with α_*DFA*_. The effect of series length on bias and variability in estimation of *H* using DFA and HG is shown in Figure 3.

**Figure 4:**
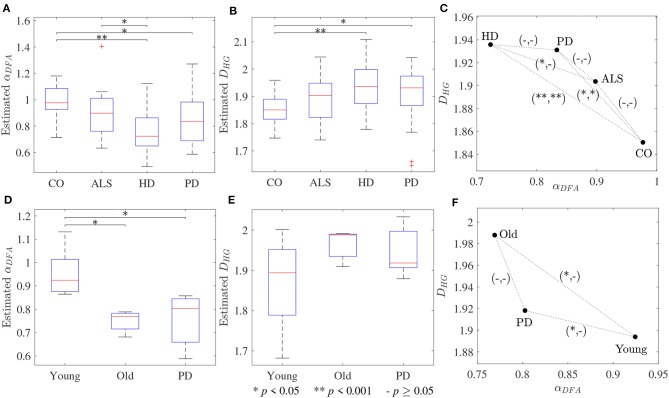
Box plots of **(A,D)** scaling exponents α_*DFA*_ and **(B,E)** fractal dimensions *D*_*HG*_; and **(C,F)** scatter plots between scaling exponents α_*DFA*_ and fractal dimensions *D*_*HG*_ for the first dataset (upper panels) and the second dataset (lower panels). The notation (−/*/**, −/*/**) denotes statistically significant difference at *p < 0.05, **p < 0.001, or no (−) in the (α_*DFA*_, *D*_*HG*_)-plane.

**Figure 5:**
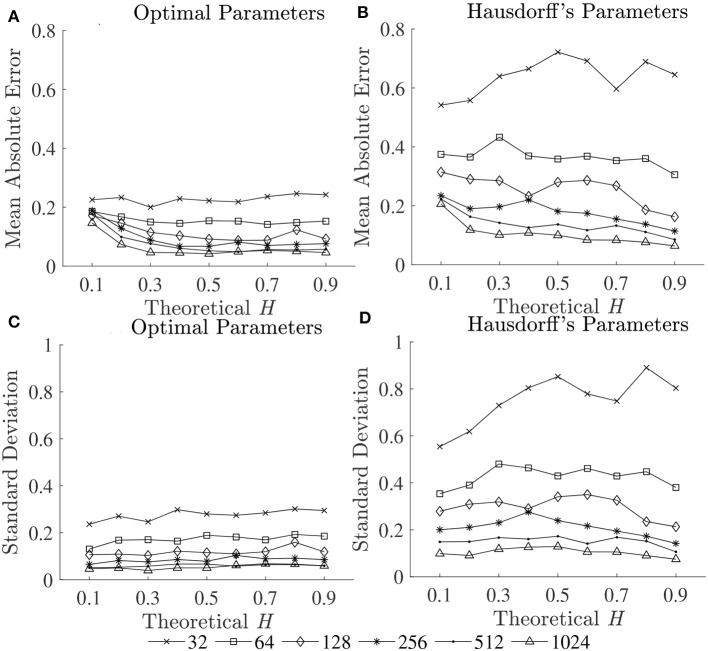
Mean errors (upper panels) and standard deviations (lower panels) of estimated Ĥ using **(A,C)** optimal parameters and **(B,D)** Hausdorff's parameters.

**Figure 6:**
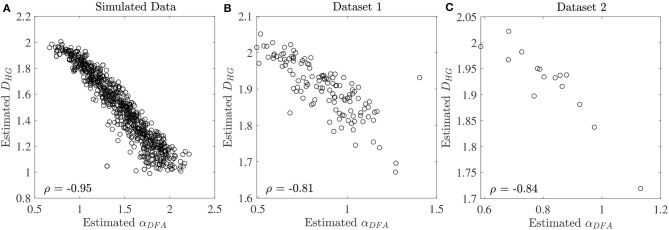
Correlation coefficients ρ between estimated α_*DFA*_ and *D*_*HG*_ at the series length of 128 data points for **(A)** the simulated fBm dataset, **(B)** the first experimental dataset, and **(C)** the second experimental dataset.

The scaling exponent α_*DFA*_ and fractal dimension *D*_*HG*_ extracted from both experimental datasets are shown as box plots in Figure 4. For the first dataset, the α_*DFA*_ were 0.98 ± 0.12, 0.89 ± 0.17, 0.76 ± 0.17, and 0.86 ± 0.19, and the *D*_*HG*_ were 1.85 ± 0.05, 1.89 ± 0.08, 1.94 ± 0.09, and 1.91 ± 0.10 for the CO, ALS, HD, and PD subjects, respectively. Significant differences among the four groups were found for both α_*DFA*_ and *D*_*HG*_ (*p* < 0.05). For the second dataset, the α_*DFA*_ were 0.95 ± 0.11, 0.75 ± 0.04, and 0.75 ± 0.12, and the *D*_*HG*_ were 1.87 ± 0.12, 1.96 ± 0.04, and 1.95 ± 0.06 for the young, elderly, and PD subjects, respectively. Significant differences among the three groups were also found for both α_*DFA*_ and *D*_*HG*_ (*p* < 0.05). Mean errors and standard deviations of α_*DFA*_ estimated using the current study's optimal parameters (Table 2) and the parameters used in the original works of the datasets (10 ≤ *n* ≤ 20) (Hausdorff et al., 1997, 2000) were computed as shown in Figure 5. Further, correlation coefficients ρ between α_*DFA*_ and *D*_*HG*_ computed over the simulated, the first, and the second experimental datasets are equal to −0.95, −0.81, and −0.84 (*p* < 0.05) as shown in Figure 6.

## 4. Discussion

### 4.1. Robust Implementation

The first purpose of this study was to identify a robust implementation of three fractal methods: DFA, BC, and HG, using short simulated time series with known *H* ranging from 0.1 to 0.9. Our recommendation for each fractal method and time series length is shown in Table 2. Although there is some variability in the optimal parameters from one series length to another, some common values were determined. For DFA, the minimum box size should be four, which is in agreement with recommendations for several data types including stride interval (e.g., Ma et al., 2005; Phinyomark et al., 2011; Wallot et al., 2013; Wiltshire et al., 2017; Ducharme et al., 2019). The maximum box size is equal to a half of the series length for very short time series (*N* ≤ 128) while an inverse relationship between the maximum box size and the series length was found for less short time series (256 ≤ *N* ≤ 1, 024). The results of the present investigation are in partial support of Damouras et al. (2010) who suggested that the maximum box size of DFA should be set to *N*/9 based on an evaluation of stride interval time series with a minimum length of 256. In the present study, this maximum box size is an optimal value for the series length of 512 data points. Using the optimal box size increment, the number of box sizes ranges from 3 (*N* = 16) to 25 (*N* = 1, 024). For consistency and simplicity, the use of half of the series length for the maximum box size and a geometric progression for the increment method is suggested (see Table S2 for the global optimal parameters across tested short time series lengths). Also, both linear and quadratic detrending methods for DFA were able to yield reasonably accurate results. For the fractal dimension estimation methods, the common values of the BC parameters were *n*_*min*_ = 4, *n*_*max*_ = *N*/2, using the arithmetic progression method with *d* = 2, while for HG, the common optimal maximum interval length was found to be four. This optimal *n*_*max*_ of the HG algorithm is consistent with several studies investigating short time series. For example, a clear discrimination between *D*_*HG*_ extracted from heart rate variability time series between healthy and diabetic subjects was found when *n*_*max*_ was in a range of between 4 and 6 (Gomolka et al., 2018).

In support of our hypothesis, the optimal implementation of the fractal methods could reduce bias and variance in the estimation of scaling exponent-like quantities and fractal dimension, and thus could provide acceptable results for shorter time series. The optimization of parameters is very important as clearly shown in Figure 1, where huge biases were found when using the worst-case parameters. For DFA and BC, these errors could be caused by an insufficient number of box sizes, an unbalanced density of points along the *x*-coordinate, or deviations from linearity occurred for smaller and larger box sizes (Peng et al., 1995; Hu et al., 2001; Damouras et al., 2010; Warlop et al., 2017). However, even when the optimal parameters were implemented, some biases were seen from the plots of theoretical *H* and mean estimated Ĥ for DFA and BC (Figure 1). It should be noted that the perfect *H* estimation should yield a straight line of slope equal to one. For HG, larger biases were observed when the maximum interval length *n*_*max*_ increased (closer to a half of the series length) (see Table S1).

### 4.2. Bias, Variability, and Series Length

The second purpose of this study was to compare the estimation accuracies of three fractal methods: DFA, BC, and HG, in terms of biases and variability. The results clearly showed that the HG algorithm is the most accurate of the three, with no apparent bias and relatively low variability (Figure 2). A systematic underestimation of Ĥ occurred when using DFA (for *H* < 0.6), while a systematic overestimation of Ĥ, conversely, occurred for BC (for *H* < 0.6). A slight overestimation and underestimation were, respectively found for DFA and BC for *H* > 0.6. The series length tended to have an influence on the magnitude and standard deviations of these biases, particularly for the shortest lengths (*N* < 128). The opposite directions and different magnitudes of the biases for DFA and BC may shed some light onto the finding of Dierick et al. (2017) which found no correlation between α_*DFA*_ and *D*_*BC*_ (ρ = −0.03, *p* = 0.72) and concluded that α and *D* can be considered as independent parameters for stride interval time series. Specifically, the fact that no linear relationship has been found between these two parameters, even though stride interval time series has been shown to exhibit self-similarity, may be due to the estimation accuracies of the implemented methods and their parameters used, rather than the walking conditions themselves. In this study, no, or weak, correlations were found between α_*DFA*_ and *D*_*BC*_ using both the first dataset (ρ = 0.08) and the second dataset (ρ = −0.37), whereas very strong correlations were found between α_*DFA*_ and *D*_*HG*_ (see section 4.3 for further discussion).

The second purpose of this study also included establishing guidelines for the selection of the length of the time series. As can be seen for all the methods, both biases and variability increased as series length decreased, whatever the *H* values (Figures 2, 3A,B). This increase was dramatic for the shortest series (*N* < 128). Based on the literature, one should expect differences in α (or *D*) of more than 0.1 between healthy and pathological gait (Hausdorff et al., 2000), therefore a mean absolute error and a standard deviation of < 0.05 is a prerequisite for estimating α (or *D*) with 0.1 error (Delignieres et al., 2006; Damouras et al., 2010). Previous studies usually considered only standard deviation, as the focus of the studies was to identify differences between groups of interest (i.e., a small standard deviation is essential while a limited and systematic bias could remain acceptable). Using the robust implementation outlined here, one could thus consider sample lengths of *N* = 128 as an acceptable level for DFA, *N* = 64 for BC, and *N* = 32 for HG (Figure 3D). However, when the aim is also to interpret the α (or *D*) values, bias should be limited as possible. If an acceptable level is 0.05, the sample length of more than 1,024 is necessary for both DFA and BC while the mean absolute value reached unacceptable levels for sample lengths less than 128 points for HG (Figure 3C). This finding suggests that *D*_*HG*_ could be calculated in a short time series containing as few as approximately 100–200 data points. This result is in support of previous studies investigating the utility of the HG algorithms for other types of data (e.g., Higuchi, 1988; Klonowski, 2007). In support of our hypothesis, more effective fractal dimension methods like HG could provide more accurate results (i.e., lower bias and variability) than Hurst exponent methods such as DFA, particularly for short time series, and could be considered as a more efficient solution to study short stride interval time series for older adults and clinical populations. In fact, even subjects are capable of walking continuously for prolonged periods of time, the properties of the long stride interval time series and the walking conditions under study may be altered because of fatigue (prolonged walking). Moreover, it is often difficult to find very long hallways for experiments and thus subjects may have to walk back and forth many times. As a result, many outliers corresponding to the turning points could be introduced. Hence, care must be taken when selecting the sample length (walking duration) in the investigation of gait variability.

### 4.3. Scaling Exponent and Fractal Dimension

The third purpose of this study was to provide guidelines for the selection of the fractal methods for analyzing stride interval time series. In the present investigation, a re-examination of two popular stride interval time series datasets was performed (Figure 4). While significant differences among multiple groups were found for both fractal methods and datasets, significant differences between pairs of groups (*post hoc* comparisons) were slightly different. Specifically, for both α_*DFA*_ and *D*_*HG*_, significant differences were found between controls and subjects with PD as well as between controls and subjects with HD. While significant differences were found between subjects with ALS and HD for α_*DFA*_, this was not found for *D*_*HG*_. Similarly, while significant differences were found between younger and older subjects as well as between young subjects and subjects with PD, these differences were not found for *D*_*HG*_. The better discrimination performance of DFA over HG in some cases might be explained by two reasons. One is a systematic bias of DFA (as observed in Figures 1A, 2A). That is, an overestimation (over 0.7) emphasizes higher exponents while an underestimation (under 0.7) emphasizes lower exponents, which presumably contributes to discriminating normal gait and alternations. The other reason is the detrending operation implemented for DFA. A quadratic detrending method was applied for these analyses and could remove constant and linear trends (non-stationarities) in the time series. Any research that focuses only on discriminating fractal features between experimental groups could therefore consider the use of DFA. Conversely, if the aim of the study is to obtain an accurate estimation of the fractal features that characterize the system under study, HG is recommended. It is important to note, however, that these analyses of experimental data were based on series lengths of 128 stride intervals (around 2–3 min walk). For an analysis of even shorter time series, HG is suggested for both mean comparison and precise estimation, as DFA could not provide the same mean values across different short series lengths (see Figure S1) and very high biases and variance were introduced for very short time series (Figure 3).

A very strong (negative) linear relationships between α_*DFA*_ and *D*_*HG*_ (ρ > 0.8) was found using both simulated and experimental data using the proposed robust implementation (Figure 6). The same degree of association between α_*DFA*_ and *D*_*HG*_ has been found in other types of data such as electroencephalogram (EEG) time series (França et al., 2018). These findings lead to the conclusion that for stride interval time series, the scaling exponent is directly related to the fractal dimension. Scaling exponent and fractal dimension quantify global and local fractal characteristics, respectively. For stride interval time series, therefore, the local properties are reflected in the global properties. Based on the associations observed in Figure 6 and the literature (Gomolka et al., 2018), a random motion (white noise) was found when α_*DFA*_ ≃ 0.5 and *D*_*HG*_ ≃ 2.0 and a more predictable, strongly auto-correlated, time series when 0.5 < α_*DFA*_ ≤ 1.0 and 1.8 ≤ *D*_*HG*_ < 2.0. However, it is worth mentioning that either α_*DFA*_ or *D*_*HG*_ might be restricted to specific walking conditions (Dierick et al., 2017), and evaluating the properties of both fractal analysis methods may provide additional information (Figures 4C,D) (Dierick et al., 2017; Croce et al., 2018).

In support of our hypotheses, and consistent with previous literature (Hausdorff et al., 1997, 2000; Kobsar et al., 2014), scaling exponents and fractal dimension showed that normal human walking (CO and Young groups in Figure 4) produced persistent stride time fluctuations and a drift toward randomness was observed with aging and neurological disorders (ALS, HD, PD, and Old groups in Figure 4). As the parameter values used for DFA in the present investigation and the original works of Hausdorff et al. (1997, 2000) are different, there are some differences between the results of both studies. For example, no significant difference between subjects with ALS and HD was found in the original work of Hausdorff et al. (2000), but a statistical significance was found in the present study. However, mean absolute errors and standard deviations using the current study's optimal parameters were lower than those of Hausdorff's parameters (Figure 5). These results suggest that variability observed in fractal dynamics of gait in the literature could be the result of different parameters used, and care must be taken when selecting values of parameters for fractal analysis methods. Future studies should also consider reporting the parameters used and their estimation accuracies to gain a greater understanding of human gait alterations with aging and disease.

### 4.4. Limitations and Future Studies

Limitations to the current research study are acknowledged. First, we chose to focus our attention on the most commonly used method in the field, DFA, and two other fractal dimension methods, BC and HG, that have been previously proposed for the study of short time series. There are other Hurst exponent and fractal dimension methods that provide accurate and low variability of exponent estimation, but for which the effect of the short time series length has not yet been investigated, such as the autoregressive fractionally integrated moving average (ARFIMA) model (Roume et al., 2019). Future studies should investigate the utility of such fractal methods for shorter time series and compare their performance with HG.

Second, simulated time series from *H* = 0.1 to *H* = 0.9 were employed as this range of values has been commonly used in most previous related studies (Delignieres et al., 2006; Roume et al., 2019). However, future research should consider incorporating a range of *H* values around the 1/*f* boundary (between 0.9 and 1.1) to gain a greater understanding of issues related to fGn/fBm classification and continuity around the boundary (Roume et al., 2019).

Third, to ensure meaningful estimates, it is necessary to identify a time series as either being fBm or fGn before selecting a relevant fractal analysis method (Eke et al., 2002). Unfortunately, it is difficult to discriminate between fBm and fGn series when values are around the 1/*f* boundary. An important distinction is that fBm processes are non-stationary, whereas fGn processes are stationary. Fairley et al. (2010) found that experimental stride interval time series are often non-stationary, and so for consistency with the pioneering works of Hausdorff and colleagues, and to simplify the interpretation, simulated fBm time series were used in this study. Moreover, the results of our own preliminary study on simulated fGn time series were in support of the current recommendations for parameters, series length, and methods. Nevertheless, future studies involving simulated fGn time series should be explored.

Finally, it is worth noting that additional techniques may be able to improve the accuracy and reduce the variability of the original methods for short stride interval time series, such as using overlapping windows or evenly spaced versions of DFA (Almurad and Delignières, 2016; Warlop et al., 2017).

## 5. Conclusions

In conclusion, the present study identified robust implementations of three fractal methods, namely DFA, BC, and HG. The importance of parameter optimization was shown, and suggests that the conflicting results in fractal analysis of stride interval time series may be due in part to the parameters used and their estimation accuracies. Using a robust implementation with optimal parameters, the estimation performance was improved producing acceptable results for shorter time series. In addition, more effective fractal dimension methods like HG could provide more accurate estimation of fractal features as compared to Hurst exponent methods like DFA. While fractal features extracted from both methods, scaling exponent and fractal dimension, are related, DFA is recommended when comparing fractal features between groups (i.e., a discriminative method) whereas HG is recommended for estimating fractal features when interpreting fractal properties (i.e., an accurate method) using series lengths of at least 128 stride intervals (around 2–3 min walk). If the aim of studies is to perform both mean comparison and precise interpretation of fractal features, HG is recommended. The present study showed the applicability of fractal analysis methods to study gait variability in older adults and clinical populations who are capable of walking continuously for at least 2–3 min.

## Data Availability Statement

The datasets analyzed for this study can be found in the Gait in Neurodegenerative Disease Database (https://doi.org/10.13026/C27G6C) and the Gait in Aging and Disease Database (https://doi.org/10.13026/C2C889).

## Author Contributions

The study design, analysis, and writing were conducted in collaboration by AP, RL, and ES.

### Conflict of Interest

The authors declare that the research was conducted in the absence of any commercial or financial relationships that could be construed as a potential conflict of interest.
