# Supplementary material for: Fractal Analysis of Human Gait Variability via Stride Interval Time Series
Source: Front Physiol. 2020 Apr 15;11:333. doi: 10.3389/fphys.2020.00333 (PMC7174763; doi:10.3389/fphys.2020.00333)
Supplement: Supplementary file 1 [file Data_Sheet_1.PDF]

# Supplementary Material

The values of the worst-case parameters for each series length were determined, in which the maximum estimation errors were found, and are shown in Table S1. The (global) optimal values of fractal parameters across lengths of the time series were also determined, in which the mean minimum estimation errors were found, and are shown in Table S2. Further, Figure S1 shows stability of $\alpha_{DFA}$ and $D_{HG}$ estimates across the series lengths.

**Table S1.** A summary of the worst-case parameters for three fractal methods (DFA, BC, and HG) for each length of the fBm time series.

| **Method** | **Parameter** | **Option for Each Series Length ($N$)** | | | | | | |
|---|---|---|---|---|---|---|---|---|
| | | **16** | **32** | **64** | **128** | **256** | **512** | **1024** |
| DFA | Minimum box size ($n_{min}$) | 2 | 4 | 4 | 4 | 4 | 4 | 2 |
| | Maximum box size ($n_{max}$) | $N/4$ | $N/5$ | $N/9$ | $N/9$ | $N/9$ | $N/9$ | $N/9$ |
| | Increment method | GP | AP | AP | GP | GP | GP | GP |
| | Increment factor ($d$) | n/a | 2 | 2 | n/a | n/a | n/a | n/a |
| | Polynomial order ($k$) | 1 | 3 | 3 | 3 | 3 | 3 | 3 |
| BC | Minimum box size ($n_{min}$) | 6 | 2 | 2 | 2 | 2 | 2 | 2 |
| | Maximum box size ($n_{max}$) | $N/2$ | $N/5$ | $N/9$ | $N/10$ | $N/9$ | $N/9$ | $N/9$ |
| | Increment method | AP | GP | GP | AP | GP | GP | GP |
| | Increment factor ($d$) | 2 | n/a | n/a | 6 | n/a | n/a | n/a |
| HG | Maximum interval length ($n_{max}$) | 8 | 16 | 32 | 64 | 128 | 256 | 512 |

**Table S2.** A summary of the (global) optimal values of all parameters for the three fractal methods (DFA, BC, and HG) across tested time series lengths (from $N = 16$ to $N = 1024$).

| **Method** | **Parameter** | **Option** |
|---|---|---|
| DFA | Minimum box size ($n_{min}$) | 4 |
| | Maximum box size ($n_{max}$) | $N/2$ |
| | Increment method | GP |
| | Increment factor ($d$) | n/a |
| | Polynomial order ($k$) | 2 |
| BC | Minimum box size ($n_{min}$) | 4 |
| | Maximum box size ($n_{max}$) | $N/2$ |
| | Increment method | AP |
| | Increment factor ($d$) | 2 |
| HG | Maximum interval length ($n_{max}$) | 4 |

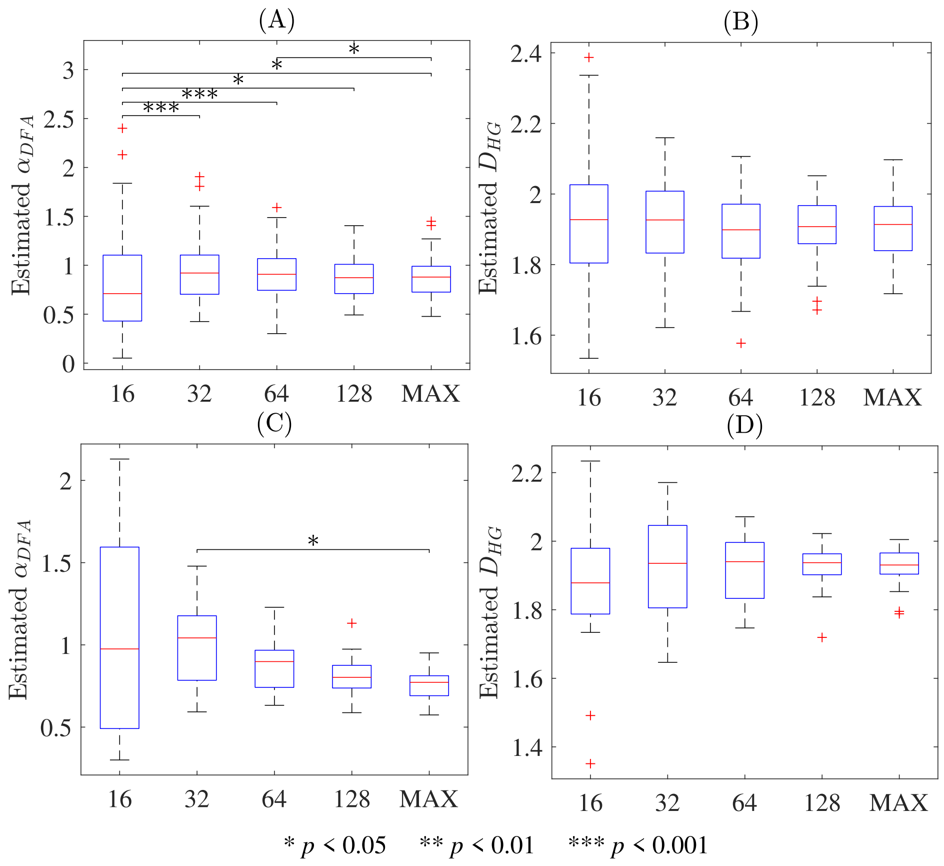


**Figure S1.** Box plots of (A, C) scaling exponents $\alpha_{DFA}$ and (B, D) fractal dimensions $D_{HG}$ averaged across subjects for the first experimental dataset (upper panels) and the second experimental dataset (lower panels).
